# Acute E-Cigarette Aerosol Condensate Exposure Disrupts the Transcriptome and Proteome Profiles of Human Bronchial Epithelial BEAS-2B Cells

**DOI:** 10.3390/cells15060525

**Published:** 2026-03-16

**Authors:** Sara Trifunovic, Jelena Kušić-Tišma, Katarina Smiljanić, Aleksandra Divac Rankov, Jelena Dinić, Mila Ljujić

**Affiliations:** 1 Institute of Molecular Genetics and Genetic Engineering (IMGGE), University of Belgrade, Vojvode Stepe 444a, 11042 Belgrade, Serbia; sara.trifunovic@imgge.bg.ac.rs (S.T.); jelena.kusic@imgge.bg.ac.rs (J.K.-T.); aleksandra.divac@imgge.bg.ac.rs (A.D.R.); 2 MEDILS—Mediterranean Institute for Life Sciences, Šetalište Ivana Meštrovića 45, 21000 Split, Croatia; 3 CoE for Molecular Food Sciences, University of Belgrade—Faculty of Chemistry (UBFC), Studentski Trg 12-16, 11158 Belgrade, Serbia; katarinas@chem.bg.ac.rs; 4 Institute for Biological Research “Siniša Stanković”—National Institute of the Republic of Serbia (IBISS), University of Belgrade, Bulevar Despota Stefana 142, 11108 Belgrade, Serbia; jelena.dinic@ibiss.bg.ac.rs

**Keywords:** electronic (e)-cigarettes, epithelial cells, mitochondria, protein modifications, protein translation, human bronchial epithelial BEAS 2B cells, acute e-cigarette exposure, lysosomes

## Abstract

The growing popularity of electronic cigarettes (e-cigarettes) necessitates a better understanding of their biological effects. In this study, we aimed to evaluate the effects of e-cigarette aerosol condensates generated from either e-cigarette carrier liquid alone or with e-cigarette liquid with nicotine and flavor on bronchial epithelial cells. BEAS-2B cells were exposed to e-cigarettes for 24 h, and transcriptional and proteomic profiling, including assessment of protein modifications, was performed. Additionally, cell-based assays were used to evaluate mitochondrial function, rate of protein synthesis, lysosomal signal, lipid droplet quantity and actin formation. Our findings reveal that short-term exposure to both types of aerosol condensates altered transcriptome and proteome profiles, disrupting cellular homeostasis in BEAS-2B cells through impaired proteostasis and mitochondrial function in response to both types of condensates. Changes in lipid and lysosome content, as well as a reduction in polymerized actin, were observed with nicotine- and flavor-containing condensate. E-cigarette exposure also induced irreversible protein modifications, including different chemical derivatives (25 out of 49 in nicotine/flavor condensate; 20 out of 48 in nicotine/flavor-free condensate; 4 out of 35 in control), suggesting their particularly harmful effect. Together, these findings point to early-onset cellular stress and impaired lung epithelial fitness caused by acute e-cigarette exposure.

## 1. Introduction

Electronic cigarettes (e-cigarettes) are battery-powered devices, originally designed to deliver nicotine in the form of aerosol and to serve as a smoking cessation tool [1]. Their use has since expanded substantially, particularly among young people and never-smokers. Liquids for e-cigarettes (e-liquids) typically contain propylene glycol (PG) and vegetable glycerin (VG) as primary solvent carriers, flavors, and may be formulated with or without nicotine [2,3]. Given the relative simplicity of e-liquid composition, the lower levels of carbon monoxide than those found in the smoke of traditional cigarettes, and the absence of tar, e-cigarettes have been considered a safer option for smokers compared to conventional combustible cigarettes. While both PG and VG have been recognized by the Food and Drug Administration as generally safe for oral use, exposure to high temperatures induces their decomposition into low molecular weight carbonyl compounds, such as the carcinogenic formaldehyde and acetaldehyde [4,5]. The addition of flavors further contributes to the diversity of compounds found in aerosols [6]. In addition to the main ingredients of e-liquid, the presence of metals and silicate particles originating from the device’s hardware was also detected in both e-liquid and aerosol [7,8]. Although the potential health consequences of e-cigarette use are still largely unknown, it has now become evident that e-cigarettes possess their own unique toxicants and may pose health risks beyond those associated with nicotine exposure.

Exposure to e-cigarettes has been shown to cause a variety of cellular effects, affect lung function and produce systemic effects across multiple organs. Lung inflammation, oxidative stress, and dysregulated repair responses have been demonstrated even after acute exposure to e-cigarette aerosol both in vitro and in vivo [9,10]. Mitochondrial dysfunction has been documented as one of the most pronounced cellular effects of e-cigarette exposure [11,12]. A first study of human lung transcriptome changes in young e-cigarette users revealed mitochondrial dysfunction and increased response to hypoxia, confirming the previous in vitro and in vivo findings [13]. Exposure to e-cigarettes has been linked with changes in the lung immunological landscape [8], structural changes in the airway epithelium [14,15,16], disruption of mucociliary clearance [17,18], reduced lung function [19,20], and increased susceptibility to infections [15]. An increasing number of studies point to systemic effects of e-cigarette exposure beyond the lungs, highlighting their complex biological impact and raising concerns about future disease risk [21,22,23,24,25].

Despite considerable advances in our understanding of e-cigarette exposure that have been made since their introduction to the market, continuing research is still needed in order to better understand the mechanisms behind the e-cigarette effect. While a lot of progress has been made in profiling the transcriptional changes of different cell types under the influence of e-cigarettes, less is known about the effect of e-cigarettes on the cell proteome, including the effect on protein modifications (PMs), leaving a critical gap in understanding early molecular events following acute exposure.

This study was designed to evaluate these early molecular signatures of acute exposure to sub-cytotoxic concentrations of e-cigarettes in the BEAS-2B cell line by transcriptional and proteomic profiling, including assessment of protein modifications. We examined condensates generated from carrier liquids alone as well as from e-liquids containing nicotine and flavoring agents, providing novel insight into early cellular stress responses, impaired proteostasis, mitochondrial dysfunction, and environmentally induced protein modifications that may precede observed toxicity. Our results demonstrate that even short-term exposure to both nicotine- and flavor-free, and nicotine- and flavor-containing aerosols alters RNA and protein expression profiles in BEAS-2B cells, disrupts protein translation and mitochondrial function, and is accompanied by protein modifications that extend beyond endogenously present signatures to include environmentally induced, irreversible changes.

## 2. Materials and Methods

### 2.1. Cells

Human normal bronchial epithelial cells BEAS-2B (cat.no. CRL-9609; ATCC, Washington, DC, USA) were cultured in Dulbecco’s Modified Eagle Medium (cat. no. D5796; Sigma-Aldrich, Taufkirchen, Germany) supplemented with 10% (*v*/*v*) Fetal Bovine Serum (cat. no. A5256701; Gibco, Waltham, MA, USA) at 37 °C in a humidified 5% CO_2_ atmosphere.

### 2.2. Preparation of E-Cigarette Aerosol Condensates

Commercially available e-cigarette liquids containing propylene glycol, glycerol, nicotine (18 mg/mL) and flavor (Virginia Tobacco flavored) (EC in further text) were purchased at the local store in Serbia. Liquid containing only propylene glycol and vegetable glycerol in a 50/50 ratio was used as a reference treatment (PG/VG in further text).

Condensates of e-cigarette liquid aerosol were generated with modifications of a previously published method [26], using a custom-made apparatus equipped with a vacuum pump. The condensation system used a 30 cm-long new tube, ensuring aerosol droplets entered the tube but did not reach the glass section of the apparatus. Condensation was facilitated by a mixture of dry ice and 96% ethanol.

For condensate generation, an A 13.5 W e-cigarette device was used, and each experimental run utilized a fully charged battery (100%) and a new coil, ensuring first-use conditions. Approximately 3–3.5 mL of e-liquid was loaded into the device, and puffing simulations were conducted with a duration of ~4 s per puff and 17 s break between puffs to avoid overheating of the e-cigarette. The apparatus was operated under vacuum for a total duration of 40 min per treatment, which included a 5 min pre-puffing phase followed by 35 min of puffing.

During the process, approximately 3 mL of e-liquid was vaporized, yielding ~500 µL of condensate. The resulting condensate consisted of a mixture of e-cigarette aerosol and moisture condensed from the air. For each e-liquid tested, a new coil and atomizer were used to avoid cross-contamination between experiments.

### 2.3. MTT Viability Assay

Cells were plated at a density of 5  ×  10^3^ cells/well in a 96-well plate, and treated 24 h after seeding with the following dilutions of e-cigarette aerosol condensate: 0.5, 1, 2, 3 and 4% (*v*/*v*). After 24 h of treatments, Thiazolyl Blue Tetrazolium Blue (MTT) (cat. no. M2128; Sigma-Aldrich, Taufkirchen, Germany) was added and incubated at 37 °C, 5% CO_2_ for 3 h. MTT formazan crystals were dissolved in dimethyl sulfoxide (DMSO) (cat. no. 20385.01; SERVA Electrophoresis GmbH, Heidelberg, Germany) and absorbance was measured at 570 nm. Samples were normalized to the untreated control.

### 2.4. RNA-Extraction, RNA-Sequencing, and Transcriptomic Analysis

BEAS-2B cells exposed to PG/VG, EC or medium alone were collected after 24 h of treatment, and total RNA was extracted using the RNeasy kit (cat. no. 74134; Qiagen, Hilden, Germany) according to the manufacturer’s recommended guidelines. Three biological replicates were used for each condition, making up a total of 9 samples. Quality and quantity of the total RNA samples were evaluated using the 2100 Bioanalyzer (Agilent, Santa Clara, CA, USA) with the RNA 6000 Nano Kit (cat. no. 5067-1511; Agilent, Santa Clara, CA, USA). All samples were considered suitable for downstream library preparation, with RNA Integrity Number (RIN) ranging from 7.7 to 9.7.

Library preparation and sequencing were carried out at Novogene Corporation Inc. (U.K., Cambridge Sequencing Center). The RNA Library was constructed according to the following workflow: messenger RNA was purified from total RNA using poly-T oligo-attached magnetic beads. After fragmentation, the first strand cDNA was synthesized using random hexamer primers, followed by the second strand cDNA synthesis. The library was ready after end repair, A-tailing, adapter ligation, size selection, amplification, and purification. Pair-end sequencing (PE150) was performed on the NovaSeq 6000 platform, generating a sequencing data volume of 32 Gb. Total reads per sample ranged from 42 to 60 M. Raw data have been deposited at NCBI’s Sequence Read Archive (SRA) and Gene Expression Omnibus (GEO) and are accessible through SRA BioProject accession number PRJNA1198719 and GEO series accession number GSE315683.

Preprocessing and quality control of raw data (fastq files) were performed by fastp v_0.23.4 with default parameters. Filtered reads were mapped to the GRCh38.p12 human reference genome utilizing STAR v_2.7.11b. Gene expression quantification was accomplished using the featuresCounts tool from subread v2.0.4 with exon feature type specified and GTF annotation file format (Homo_sapiens.GRCh38.111.chr.gtf). Multi-mapping and multi-overlapping reads were ignored. Three biological replicates were used for each condition.

Differential expression analysis across all conditions was performed using DESeq2 (v1.46.0). The batch effect was removed with ComBat-Seq from the SVA package (v_3.54.0) using the full model and the treatment variable as the group parameter. The dds object was made from the adjusted count matrix, and only genes with read counts ≥ 10 in at least 3 samples were kept. This filtering reduced the transcript pool from 63,187 to 16,100. Data normalization was executed using the size factor adjustment method in DESeq2. Differential expression analysis was performed to compare the PG/VG and the EC treatment with each other and the controls. The Wald significance method in DESeq2 was employed, with differentially expressed genes being defined by a false discovery rate (FDR) < 0.05. Log fold change (LFC) shrinkage was applied using the ashr package to minimize result variability [27]. Up- and down-regulated genes for a given pairwise comparison were subjected to functional enrichment analysis and visualized using the clusterProfiler package (v_4.12.6). To visualize shared genes between groups of DEGs, we used a Venn diagram created using the online tool Interactivenn, available at https://www.interactivenn.net/ (accessed on 20 November 2025) [28].

### 2.5. Preparation of Cells Protein Extracts for Nano Liquid Chromatography Coupled to High Resolution Tandem Mass Spectrometry (nLC-MS/MS)

BEAS-2B cells were lysed with RIPA Lysis and Extraction buffer (cat. no. 89900; Thermo Scientific, Rockwood, TN, USA), and protein concentration was determined by BCA test (cat. no. 23227; Thermo Scientific, Rockwood, TN, USA) using NanoDrop 2000c (Thermo Scientific, Rockwood, TN, USA). The extracts were diluted accordingly so all samples had the same concentration with 15 μg of proteins resolved by reducing one-dimensional sodium dodecyl sulphate-polyacrylamide gel electrophoresis (1D SDS-PAGE), to a point of entering the separating gel (maximal path up to 5 mm of 14% SDS-PA gel). Gels were stained with Coomassie^®^ Brilliant Blue R 250 (cat. no. 17525.01; SERVA Electrophoresis GmbH, Heidelberg, Germany), and for the shotgun in-gel digestion, gel bands containing all proteins were excised from the gel, reduced by 10 mM dithiothreitol (cat. no. D0632; Sigma-Aldrich, Taufkirchen, Germany), afterwards alkylated with 55 mM iodoacetamide (cat. no. I1149; Sigma-Aldrich, Taufkirchen, Germany) and finally digested with proteomics-grade trypsin (cat. no. T6567; Sigma-Aldrich, Taufkirchen, Germany) in a 1:30 enzyme to substrate ratio, overnight at 37 °C [29]. Trypsin digests were cleaned and desalted with HyperSep tips C18 (cat. no. 60109-209; Thermo Scientific, Rockwood, TN, USA), evaporated and reconstituted in 20 μL of 0.1% aqueous solution of formic acid (cat. no. PI85170; Thermo Scientific, Rockwood, TN, USA). Three biological replicates were used for each treatment, making up a total of 9 samples.

### 2.6. nLC-MS/MS

Tryptic peptides in 1 µL injection volume and with approximately 375 ng in each sample were chromatographically separated with nLC system UltiMate 3000 (Thermo Scientific, Waltham, MA, USA) using (A) 0.1% formic acid in water, and (B), 0.1% formic acid in acetonitrile as the mobile phase, using a 5–70–95% B gradient at a flow rate of 250 nL/min lasting 88 min. Peptides were analyzed using Orbitrap ExplorisTM 240 (Thermo Scientific Inc., Bremen, Germany) in the data-dependent mode with the 20 most intense precursors subjected to fragmentation by collision-induced dissociation (HCD 30%) [30]. Scan range in m/z was set to 350–1400, resolution of the full MS1 scan was 60,000 and 15,000 for MS2, the isolation window (*m*/*z*) was 1.4; normalized automatic gain control targets of MS1 and MS2 were 300% and 200%, respectively, while maximum injection time was the same for both scans, e.g., 25 ms. Dynamic exclusion was set to 3 s after the first repetition ion event.

### 2.7. Identification and Label-Free Quantification of Cell Treatments and Quantitative Protein Modifications (PM) Profiling

BEAS-2B proteins were identified with the PEAKS X Pro platform (Bioinformatics Solutions Inc., Waterloo, ON, Canada) [31] and its PTM algorithm against a UniProtKB database (http://www.uniprot.org/) of Homo sapiens species (taxon ID 9606, 42379 sequences, and accessed on 30 May 2025) and contamination database as common Repository of Adventitious Protein entries (https://www.thegpm.org/, 116 sequences, accessed on 30 May 2025). Oxidation (Met) and deamidation (Gln, Asn) were considered as variable modifications, with carbamidomethylation (Cys) set as fixed in the PEAKS DB algorithm. In the PEAKS PTM algorithm, an unrestricted protein modification (PM) search was undertaken using an available list of 313 PMs from the Unimod database. Up to 2 missed trypsin cleavages with non-specific cleavages at one end (semi-specific mode of action) of a peptide were allowed. Mass tolerances were set to ±10 ppm for parent ions and ±0.02 Da for fragment ions. Protein filters were as follows: protein −10 log*p* ≥ 20, protein unique peptides ≥ 2, and “A” Score for confident PMs identification of at least 50. Peptide filters were as follows: false discovery rate for peptide-spectrum matches <0.1%; therefore, the resulting false discovery rate of the peptide sequence was lower than 0.5%, and the de novo alignment local confidence score was ≥80%.

The quantity of identified BEAS-2B proteins was compared by label-free quantification (LFQ) using the PEAKS Q algorithm. The term “PM profiling” means the relative extent of the modifications within a single sample. Relative LFQ of proteins and PMs in 3 treatment groups with 3 biological replicates per group was done as described previously [32,33]. Briefly, in the LFQ section, cut off filter criteria for defining confidently differentially regulated proteins were a significance score of 20 (−10log*p*, *p* < 0.01) and a 2-fold change in log2 up/down ratio, comparing any of the treatments with respect to the control group.

The bioinformatics, with functional enrichment analyses and Venn diagrams, were done in Functional Enrichment Analysis Tool—FunRich software v 3.1.3 (http://funrich.org) (accessed on 30 May 2025) and Quick GO (https://www.ebi.ac.uk/QuickGO) (accessed on 30 May 2025). As a background database, a completely reviewed UniProt set of Homo sapiens species (taxon ID 9606, 20,423 sequences) was used, unless otherwise stated.

The mass spectrometry proteomics data have been deposited to the ProteomeXchange Consortium via the PRIDE [34] partner repository with the dataset identifier PXD072831 and 10.6019/PXD072831.

### 2.8. Mitochondrial Membrane Potential Analysis

Mitochondrial membrane potential was evaluated using cationic dyes JC-1 and Tetramethyl rhodamine ethyl ester (TMRE). BEAS-2B cells were seeded in 96-well plates at a density of 7000 cells per well. After 24 h, cells were treated and incubated for an additional 24 h. To measure mitochondrial membrane potential, cells were incubated with either 1 μM JC-1 (cat. no. T3168; Thermo Fisher Scientific, Waltham, MA, USA) or 500 nM TMRE (cat. no. T669; Sigma-Aldrich, Taufkirchen, Germany) added to the culture media for 15 min at 37 °C. Cells were then washed with phosphate-buffered saline (PBS) and visualized using the ImageXpress^®^ Pico Automated Cell Imaging System (Molecular Devices, San Jose, CA, USA) with a 10× objective. The obtained TMRE images were analyzed using CellReporterXpress software v2.8.2.669 (Molecular Devices, San Jose, CA, USA) using the Cell Scoring Analysis Protocol to quantify average fluorescence intensity per cell. JC-1 fluorescence was analyzed using Fiji software v1.54p (National Institutes of Health, Bethesda, MD, USA). Briefly, individual cells were segmented, background was automatically subtracted from the red (JC-1 aggregates) and green (JC-1 monomers) channels, and mean per-cell fluorescence intensities were measured to calculate the red/green fluorescence ratio. A minimum of 300 cells was analyzed per condition.

### 2.9. Protein Synthesis Assay

Detection of total protein synthesis was performed using Protein Synthesis Assay Kit (cat. no. ab273286; Abcam, Cambridge, UK) according to the manufacturer’s specifications. This assay utilizes a cell-permeable analog of puromycin, O-propargyl-puromycin (OP-puromycin), that forms covalent conjugates with nascent polypeptide chains. Truncated polypeptides are then fluorescently labelled in a copper-dependent reaction using a fluorescent azide. Cells were pre-treated with e-cigarette aerosol condensate for 24 h and incubated with protein label dye for 3 h, following the manufacturer’s instructions. Cyclohexamide was used as a negative control. Nuclei were labeled by incubating cells with Hoechst 33342 (cat. no. H1399; Thermo Fisher Scientific, Waltham, MA, USA) (1 µg/mL) for 15 min at room temperature (RT). Fluorescence images were acquired using the ImageXpress^®^ Pico Automated Cell Imaging System (Molecular Devices, San Jose, CA, USA) with a 10× objective. OP-puromycin fluorescence was analyzed by CellReporterXpress software v2.8.2.669 (Molecular Devices, San Jose, CA, USA) using the Cell Scoring Analysis Protocol to quantify average per-cell fluorescence intensities.

### 2.10. Lysosome Staining

BEAS-2B cells were plated in 96-well plates at a concentration of 10,000 cells per well.

After 24 h, the cells received treatment and were incubated for another 24 h. Lysosomes were labeled using 1 µM LysoTracker^®^ Green DND-26 (cat. no. L7526; Thermo Fisher Scientific, Waltham, MA, USA). The staining was carried out in PBS for 30 min at 37 °C. Nuclei were labeled by incubating cells with Hoechst 33342 (cat. no. H1399; Thermo Fisher Scientific, Waltham, MA, USA) (1 µg/mL) for 15 min at RT. Following staining, cells were washed with PBS, and live-cell imaging was conducted in PBS using the ImageXpress^®^ Pico Automated Cell Imaging System (Molecular Devices, San Jose, CA, USA) with a 10× objective. Fluorescent images were subsequently analyzed with CellReporterXpress software v2.8.2.669 (Molecular Devices, San Jose, CA, USA) using the Cell Scoring Analysis Protocol to quantify average per-cell fluorescence intensities.

### 2.11. Lipid Staining

BEAS-2B cells were plated in 30-mm glass-bottom dishes (cat. no. 627860; Greiner, Kremsmünster, Austria) at a density of 150,000 cells per dish. After 24 h, the cells were treated and incubated for another 24 h. Neutral lipids were stained in PBS with 5 µM BODIPY™ 493/503 (4,4-Difluoro-1,3,5,7,8-Pentamethyl-4-Bora-3a,4a-Diaza-s-Indacene) [35] (cat. no. D3922; Thermo Fisher Scientific, Waltham, MA, USA) for 20 min at 37 °C. Live cells were imaged on a Leica TCS SP8 confocal microscope (Leica Microsystems, Wetzlar, Germany) using a HC PL APO CS2 40×/1.30 objective. Lipid droplets were quantified using Fiji software v1.54p (National Institutes of Health, Bethesda, MD, USA) with the 3D Objects Counter plugin. Z-stack images were thresholded individually to segment lipid droplets. Object number and total volume (µm^3^) were obtained per field and normalized to cell number to generate per-cell values. Mean lipid droplet volume was calculated as total volume divided by object count for each field. A minimum of 350 cells was analyzed per condition.

### 2.12. Filamentous Actin Staining Analysis

BEAS-2B cells were plated on glass coverslips (cat. no. 0117500; Paul Marienfeld GmbH, Lauda-Königshofen, Germany) in 24-well plates at a density of 50,000 cells per well. After 24 h, the cells were treated and incubated for another 24 h. Cells were fixed with 4% paraformaldehyde (cat. no. J61899.AK; Thermo Scientific, Rockwood, TN, USA) for 20 min at RT and permeabilized with 0.1% Triton X-100 (cat. no. T8787; Sigma-Aldrich, Taufkirchen, Germany) for 1 min. Filamentous actin (F-actin) was stained using Alexa Fluor™ 555 phalloidin (cat. no. A34055; Thermo Fisher Scientific, Waltham, MA, USA) (1:200 dilution) for 30 min at RT in the dark. Nuclei were labeled by incubating cells with Hoechst 33342 (cat. no. H1399; Thermo Fisher Scientific, Waltham, MA, USA) (1 µg/mL) for 15 min at RT. Images were acquired on a Leica DMRB fluorescence microscope (Leica Microsystems, Wetzlar, Germany) using a 20× objective with identical exposure and gain settings for all groups. F-actin fluorescence was analyzed using Fiji software v1.54p (National Institutes of Health, Bethesda, MD, USA). Briefly, the F-actin channel was thresholded using the Otsu method to isolate filaments, and thresholded area, mean intensity, and integrated density were measured for each field and normalized per cell. A minimum of 400 cells was analyzed per condition.

### 2.13. Statistical Analyses

For data from mitochondrial membrane potential, lysosome content, lipid droplet accumulation, actin filaments and protein synthesis assays, normality was assessed using the Shapiro–Wilk test, followed by one-way ANOVA with Tukey’s post-hoc test. Results of the MTT assay were analyzed using an unpaired *t*-test with Welch correction. Statistical significance was set at *p* < 0.05 and indicated as: *p* < 0.05 (*), *p* < 0.01 (**), *p* < 0.001 (***), *p* < 0.0001 (****). Data analysis was performed using GraphPad Prism version 8 (La Jolla, CA, USA).

The PEAKS Q statistical test was used within the PEAKS Studio proteomic platform to test for significant differences in relative label-free protein quantification. The hypergeometric test, as an uncorrected *p*-value assessment method, and the Bonferroni test, together with BH and Q-value (Storey-Tibshirani method) tests as corrected versions of *p*-value assessments, were used by FunRich software version 3.1.3.

## 3. Results

### 3.1. Analysis of Differentially Expressed Genes (DEGs)

To examine the impact of e-cigarette aerosol condensate on gene expression in BEAS-2B cells, we analyzed their transcriptional profiles following a 24 h exposure to PG/VG and EC at a 2% (*v*/*v*) dilution. Untreated cells were used as a control. Prior to the experiments, cell viability was evaluated across various concentrations of PG/VG and EC (0.5, 1, 2, 3 and 4% (*v*/*v*)) using the MTT assay (Figure S1).

**Figure 1:**
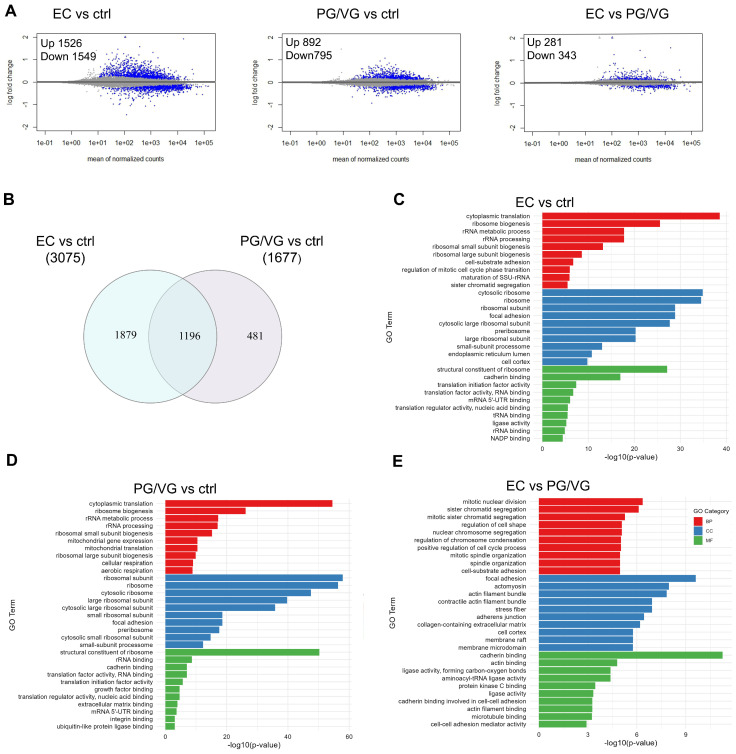
Transcriptomic analysis of BEAS-2B cells exposed to e-cigarette aerosol condensate (ctrl represents untreated cells, EC represents cells treated with e-cigarette condensate with flavor and nicotine and PG/VG carrier solution of propylene glycol and glycerol). (**A**) MA-plots for the shrunken log2fold changes (*p*adj < 0.01). (**B**) The Venn diagram illustrates the overlapping of DEGs from PG/VG and EC treatments. A Venn diagram was created using the online tool Interactivenn. (**C**–**E**) Gene Ontology (GO) enrichment analysis of differentially expressed genes (DEGs) unique to 3 different comparisons. Over-representation analysis (ORA) showing the top 10 GO terms of each ontology category—cellular component (CC), molecular function (MF), and biological process (BP). Analyses were done in R with ClusterProfiler. Three replicates were used for each condition.

The analysis of differential gene expression was performed by comparing the PG/VG and EC treatment groups with the control group, as well as comparing EC with PG/VG treatment. Three replicates were performed for each treatment and control. Differentially expressed genes (DEGs) were those with an FDR < 0.05; no log fold change (LFC) cutoff was applied to prevent bias in gene selection. In general, the exposure to EC resulted in greater variations in the expression levels compared to PG/VG (Figure 1A). There were 3075 differentially expressed genes (DEGs) between the EC and the control group, consisting of 1526 upregulated and 1549 downregulated genes; 1677 between PG/VG and the control group, with 892 downregulated and 785 upregulated. There were only 624 DEGs between EC and PG/VG group, 281 upregulated and 343 downregulated (Table S1). A Venn diagram of the DEGs identified in the EC vs. control and PG/VG vs. control groups revealed that 1196 genes were differentially expressed in both treatment conditions (Figure 1B). In our analysis, only one gene (*ARRDC3*) had [LFC]  >  1 in the PG/VG vs. control comparison, and 54 in the EC vs. control comparison. In the EC vs. control comparison, there were 54 genes with [LFC]  >  1, 6 downregulated and 48 upregulated. In the EC vs. PG/VG comparison, there were 9 upregulated genes with [LFC]  >  1.

**Figure 2:**
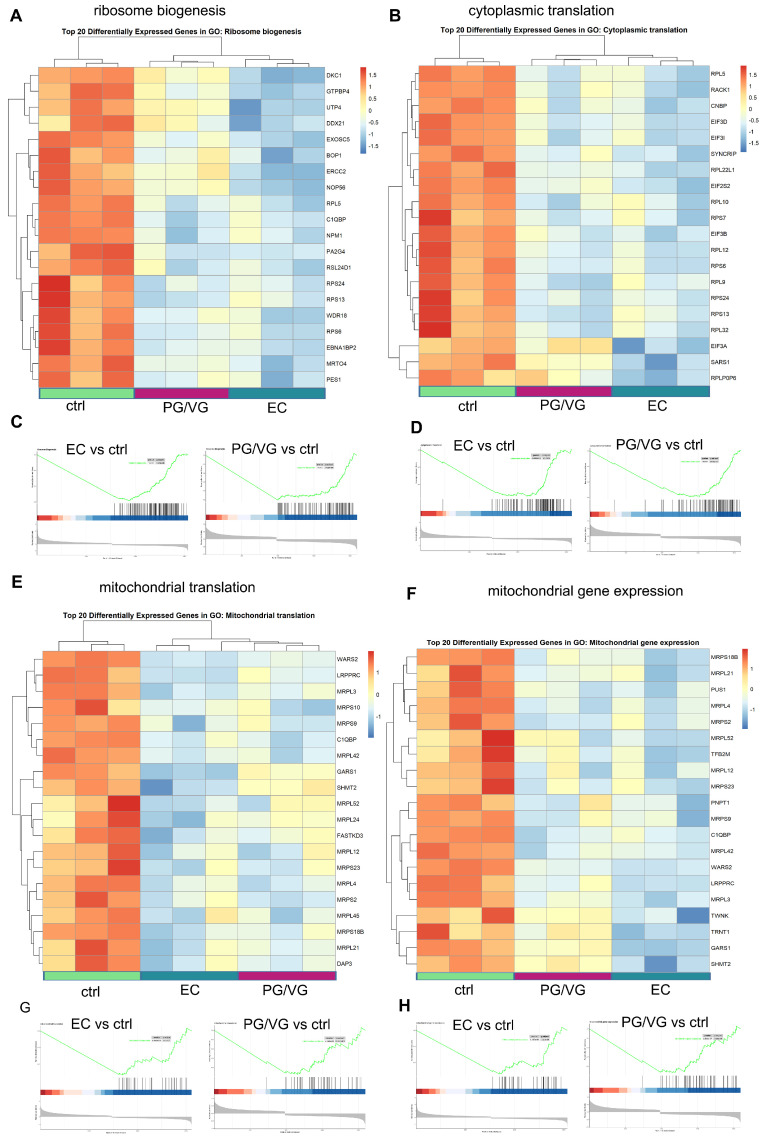
Impacts of E-Cigarette Exposure on Translation and Mitochondrial Gene Expression in BEAS-2B Cells. (**A**,**B**) Heatmaps for the top 20 differentially expressed genes belonging to ribosome biogenesis (**A**) and cytoplasmic translation (**B**) gene sets. (**C**,**D**) Enrichment plots of ribosome biogenesis (**C**) and cytoplasmic translation (**D**). (**E**,**F**) Heatmaps for the top 20 differentially expressed genes belonging to mitochondrial translation (**E**) and mitochondrial gene expression (**F**) gene sets. (**G**,**H**) Enrichment plots of mitochondrial translation (**G**) and mitochondrial gene expression (**H**) gene set in BEAS-2B cells exposed to e-cigarettes. The expression level of each transcript in the heatmaps is represented by a color, ranging from blue (low) to red (high). The green line in enrichment plots indicates the trend of the enrichment score (ES), with a negative enrichment score corresponding to downregulation of the shown processes. Three replicates were used for each condition.

Gene Ontology enrichment analysis was performed using overrepresentation analysis via enrichGO to identify the most significantly altered gene ontologies associated with DEGs in EC vs. control, PG/VG vs. control, and EC vs. PG/VG comparisons. In both the EC vs. control and PG/VG vs. control comparisons, the top 3 enriched biological processes were related to cytoplasmic translation (GO:0002181), ribosome biogenesis (GO:0042254), and rRNA metabolic process (GO:0016072) (Figure 1C–E). Additionally, ribosomes (GO:0005840) and structural constituent of ribosome (GO:0003735) were among the most significantly enriched molecular function terms in both comparisons. Gene Set Enrichment Analysis (GSEA) further revealed that the pathway associated with translation was suppressed in both EC and PG/VG-treated cells compared to the control (Figure S2), indicating the negative impact of these treatments on protein synthesis. Among the top 20 ranking genes included in the cytoplasmic translation process were *EIF3A*, *EIF3B*, *EIF3I* and *EIF3D*, which code the subunits of eukaryotic initiation factor 3 (eIF3) as well as *RPL5* and *RPS6* genes coding the structural components of the ribosome (Figure 2A,B). None of these genes had [LFC]  > 0.5, indicating subtle changes in their expression. The enrichment plots illustrating the downregulation of the transcriptome involved in the processes of ribosome biogenesis and cytoplasmic translation in treatments are given in Figure 2C,D.

The first ten biological processes in PG/VG vs. control comparison also included mitochondrial gene expression (GO:0140053) and mitochondrial translation (GO:0032543), as well as aerobic respiration (GO:0009060) and cellular respiration (GO:0045333), indicating changes in mitochondrial function (Figure 1D). Enrichment plots for mitochondrial gene expression and mitochondrial translation gene sets revealed downregulation of these processes in both EC and PG/VG treatments (Figure 2G,H), while aerobic respiration and cellular respiration were not significantly affected in EC treatment. Downregulated genes in both treatments included genes coding for mitochondrial ribosome proteins that are encoded by the nuclear genome, such as *MPRS18B*, *MRPL3*, *MRPL21*, *DAP3* and *MRPL4* (Figure 2E,F). *COA3*, a gene that codes for Cytochrome c Oxidase Assembly Factor 3, and *ND2*, a mitochondrial gene that encodes subunit 2 of NADH dehydrogenase, were slightly downregulated in PG/VG compared to the control, but were not among DEGs in EC-treated cells (Table S1).

Lipid metabolic process (GO:0006629) was upregulated in EC treatment when compared to both control and PG/VG treatment, with upregulated genes including *NEU1*, which is involved in the metabolism of glycolipids and lysosomal catabolism, and cytochrome P450 genes *CYP1B1* and *CYP1A1*, among others (Table S1)*. CYP1B1* and *CYP1A1* were among the most upregulated genes in EC treatment, when compared to untreated cells and also when compared to PG/VG-treated cells (Table S1). *CYP1B1* was found to be upregulated by CS exposure and plays a key role in CS-induced lipid accumulation in alveolar type 2 cells [36]. *ABCA7* and *ABCA3*, involved in the transport of phospholipids, were slightly upregulated in both EC and PG/VG compared to the control. *BCAT1*, coding for a key enzyme in branched amino acid metabolism, whose increased transcription is associated with poor survival of lung cancer patients, was downregulated in EC treatment [37].

When EC was compared to PG/VG, cellular responses to chemical stimulus and hormone metabolic process were activated, while enriched cellular components included focal adhesion (GO:0005925), actin filament bundle (GO:0032432) and membrane domain (GO:0045121). There was enrichment in the biological processes of mitotic nuclear division (GO:0140014) and sister chromatid segregation (GO:0000070) (Figure 1E), with GSEA revealing suppression of these processes in EC treatment compared to PG/VG (Figure S2). Downregulated genes related to cytoskeleton organization included *FLNB*, *FLNA*, *FBLIM1*, *RHOA* and *DLC1*, among others. Inhibition of RhoA and disruption of intercellular adhesion molecules have been implicated in the effect of cigarette smoke extract on lung vascular cells’ permeability [38]. Transmembrane signaling receptor activity (GO:0004888) and molecular transducer activity (GO:0060089) were among the most activated processes in EC vs. control, while plasma membrane (GO:0005886) and cell surface receptor binding (GO:0005102) were the most upregulated in PG/VG vs. control. These could reflect changes in cell–cell interaction and membrane trafficking. Additionally, positive regulation of cytokine production (GO:0050715) and response to virus (GO:0009615) were observed, suggesting that upregulation of plasma membrane components could also be to promote immune response.

Genes that were upregulated in EC-treated cells when compared to the control, with the [LFC]  >  1, included those linked with inflammatory response (*IL24*, *SECTM1*, *RSAD2/MX2*, *DMBT1*), xenobiotic metabolism (*CYP1A1*, *CYP1B1*, *TIPARP*, *ALDH3A1*) and cytoskeleton organization (*VCAN*, *NGFR*). *POSTN*, coding for a secreted extracellular matrix protein, was among the downregulated DEGs with [LFC]  >  1 in EC vs. control comparison. Out of 48 genes upregulated in EC when compared to the control that had [LFC]  >  1, 7 genes were also upregulated when EC was compared to PG/VG (*IL24*, *TIPARP*, *SECTM1*, *VIPR1*, *SHISA2*, *CYP1B1*, *CYP1A1*).

### 3.2. Effect on BEAS-2B Proteome

The shotgun proteomic data from control and treatment groups in triplicate were used to visualize trends in GO enrichment categories and significantly differentially expressed proteins (DEPs) (Table S2).

**Figure 3:**
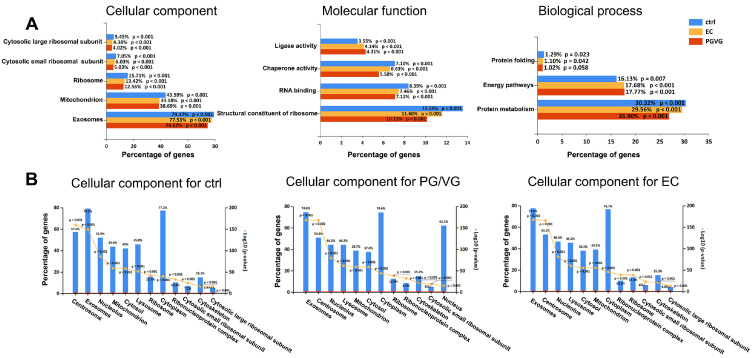
Proteomics-Derived Gene Ontology Profiling of BEAS-2B cell proteome following EC and PG/VG treatments. (**A**) Comparison of cellular component (CC), molecular function (MF), and biological process (BP) among BEAS-2B treatments was performed using FunRich 3.1.3 software. Statistically significant enrichment of gene products is presented for representative examples in each GO category; *p*-values were calculated using the hypergeometric test and corrected for multiple comparisons using the Bonferroni method. (**B**) Enrichment analysis of cellular component categories in BEAS-2B proteomes following EC and PG/VG exposure. Cellular component profiling and enrichment analysis were performed for BEAS-2B cells under each treatment condition. The legend indicates −log_10_(*p*-value) as the probability score from gene ontology enrichment analysis. *p*-values were calculated using the hypergeometric test and corrected for multiple comparisons with the Bonferroni method. All analyses and graphics were generated using FunRich 3.1.3 software. Three biological replicates were used for each condition.

Protein metabolism appears impaired in both treatments (less enriched), as indicated by the decrease in related cellular component and molecular function categories, particularly those associated with ribosomes and their function (Figure 3A). Another notable aspect of GO enrichment that differs from the control is energy metabolism. Energy pathways are additionally enriched in the treatments, while the mitochondrion category within cellular components shows the opposite trend. This may reflect an increase in alternative energy pathways not directly linked to mitochondria (e.g., glycolysis), or a compensatory effect due to reduced mitochondrial components (Figure 3A). Chaperone activity was impaired in both treatments compared to the control, as visible in Figure 3A under molecular function and biological processes for EC and PG/VG conditions, which is also in accordance with the downregulation of genes responsible for chaperone-mediated protein folding (*CCT3*, *CCT7*, *CCT8*, *CCT5*) as evidenced by transcriptome analysis (Table S1).

Some of these observations are mirrored in the FunRich software’s automated selection of the most significantly enriched cellular component categories, as reflected in their order of appearance (Figure 3B). For example, ribosomes rank seventh, eighth, and ninth in control, PG/VG, and EC, respectively. Similarly, mitochondria rank fourth, fifth, and sixth in control, PG/VG, and EC, respectively. Notably, the nucleus appears in PG/VG as the twelfth category, while it is absent from the top 12 most significantly enriched categories in both control and EC. The proteomic, label-free quantitative findings of differentially expressed proteins (DEPs) (Table S2) mostly support the qualitative enrichment trend of representative categories in the cellular component, biological process, and molecular function aspects (Figure 3A). The set of 35 DEPs was analyzed using QuickGO (https://www.ebi.ac.uk/QuickGO) (accessed on 30 May 2025), applying the same GO terms as presented in Figure 2A. For instance, under the GO term “ribosome” (GO:0005840), two proteins were annotated as “part_of” the ribosome and were both downregulated across treatments. Specifically, 60S ribosomal protein L34 (RPL34) was significantly downregulated in EC, while 40S ribosomal protein S11 (RPS11) was significantly downregulated in PG/VG, reinforcing the observed decreasing trend in ribosome-related enrichment. These findings are consistent with the downregulation of translation pathways and mitochondrial gene expression found in transcriptome analysis. Proteome analysis also revealed enrichment of cellular components for lysosome in treatments where it was ranked fourth in both EC and PG/VG, while it occupied sixth position in the control.

When analyzing biological process generation of precursor metabolites and energy (GO:0006091), 2 DEPs were identified: isoform 2 of serine/threonine-protein phosphatase PP1-gamma catalytic subunit, which was upregulated in both treatments, and glyceraldehyde-3-phosphate dehydrogenase, which was significantly upregulated only in EC. Analysis of protein metabolism (GO:0019538, biological process) revealed that all 3 DEPs associated with translation, a child term of protein metabolism, were significantly downregulated in both treatments compared to the control: RPL34, RPS11, and cytoplasmic asparagine–tRNA ligase, with expression ratios of Ctrl:PG/VG:EC = 1.00:0.77:0.47, 1.00:0.39:0.86, and 1.00:0.62:0.44, respectively (Table S2).

Interestingly, all proteins associated with proteolysis (GO:0006508, a child term of protein metabolism) were upregulated at least in one treatment: isoform 3 of leukotriene A4 hydrolase (1.00:2.19:0.97); isoform 2 of cytosol aminopeptidase (1.00:2.13:1.28); 26S proteasome regulatory subunit 7, involved in the ubiquitin-dependent protein catabolic process (1.00:2.06:1.75); ATP-dependent Clp protease proteolytic subunit, mitochondrial, which plays a role in protein quality control by degrading misfolded or incompletely synthesized proteins (1.00:0.90:6.43); and eIF-2-alpha kinase activator GCN1, which is involved in protein–RNA covalent cross-link repair and the rescue of stalled ribosomes (1.00:2.48:1.07). Collectively, these proteins are associated with cellular repair mechanisms, suggesting an increased level of molecular damage in the treatments.

Coming to the molecular function aspect, the situation with ribosomes and translation is replicated, since the same set of DEPs emerges when employing appropriate GO terms (GO:0003723-RNA binding and GO:0003735-structural constituents of ribosomes).

Finally, some very interesting examples of DEPs upregulated in EC treatment, which do not fall into the above-mentioned GO terms, are tyrosine-protein kinase JAK3, and Di-Ras2, a small GTP-binding protein that acts as a molecular switch in cell signalling pathways, and can activate the RAF/MEK/MAPK pathway. Similarly, isoform 2 of Serine/arginine-rich splicing factor 2 protein (SRSF2) was also found to be upregulated in EC and has been reported in studies with aging lung mucosa [39] or with apoptotic lung cells [40].

To further distinguish the effects of nicotine and flavor-containing e-cigarette aerosol from the PG/VG carrier, we compared EC directly to PG/VG. Recalculation of 35 DEPs in Table S2 identified 21 differentially expressed proteins between these two treatments, with 12 upregulated and 9 downregulated in EC. Ten of the upregulated DEPs (DIRAS2, MYH9, ACTN1, NONO, NARS1, H2AC6, H1-4, RPL34, PA2G4, MCM3) were also consistently upregulated in EC vs. control, while two (TOP2A and LAP3) showed relative upregulation in EC compared to PG/VG despite not reaching the cutoff in EC vs. control. Of the downregulated DEPs, five followed the control trend, whereas four (MYH13, H3C1, H1-5, SF3B3) behaved like the control in EC but were upregulated in PG/VG. These findings highlight nicotine and flavor-specific proteomic effects distinct from the PG/VG carrier.

### 3.3. Protein Modifications

**Figure 4:**
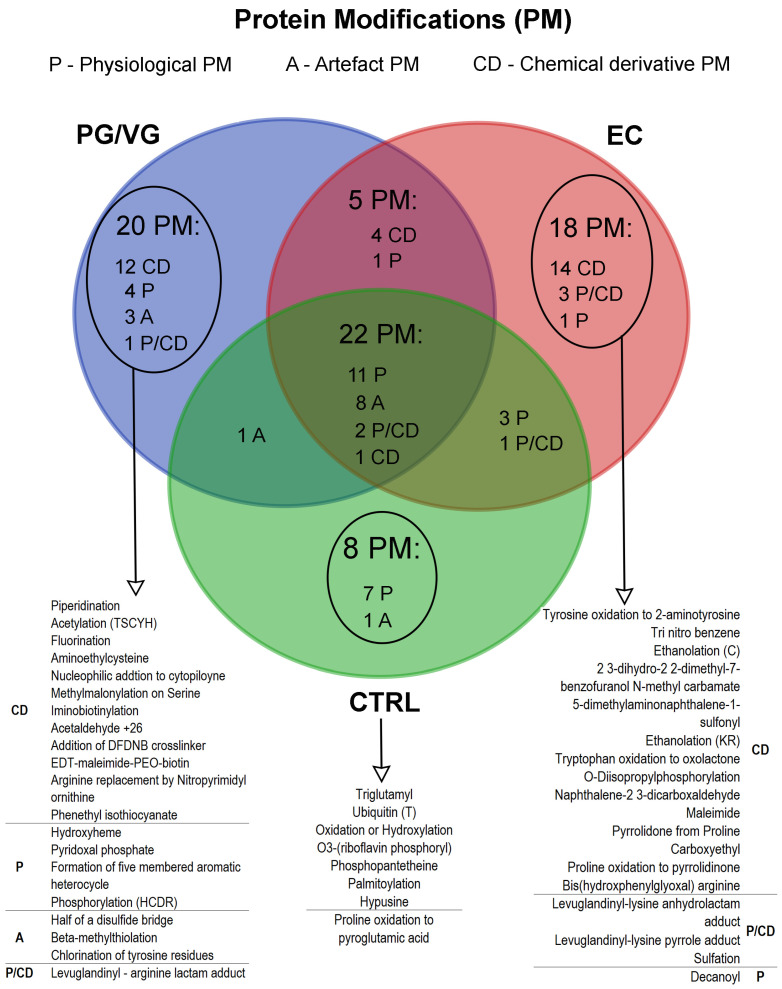
Venn diagram of protein modification (PMs) profiling in BEAS-2B cells exposed to e-cigarette aerosol condensate. CD—chemical derivative modification, P—physiological modifications (posttranslational modification (PTM) or PTM-like protein modification), A—artefact protein modification. The Venn diagram was created using FunRich software (http://funrich.org).

Protein modification profiles of each triplicate enabled both qualitative and quantitative pattern assessment of post-translational and chemical modifications caused by environmental factors, collectively abbreviated here as PMs (Figure 4, Table 1). Analysis of PMs in BEAS-2B cells treated with e-cigarette condensate revealed that EC-treated cells exhibited the highest number of different chemical derivative (CD) protein modifications (25 out of 49), followed by PG/VG treatment with 20 out of 48, while the control contained only 4 CD modifications out of 35. This trend is mirrored in the distribution of unique CD modifications: 78% (14 out of 18) in EC-treated cells and 60% (12 out of 20) in PG/VG-treated cells, with none detected in the control (Figure 4).

As shown in Table 1, the EC group contains seven PMs classified as toxic, while the PG/VG group has four, and the control group does not exhibit any PMs of this classification. Toxic PMs found in EC treatment comprise trinitrobenzene, 2,3-dihydro-2,2-dimethyl-7-benzofuranol N-methyl carbamate (carbofuran), bis(hydroxyphenylglyoxal) arginine, 5-dimethylaminonaphthalene-1-sulfonyl, O-diisopropylphosphorylation, N-Succinimidyl-2-morpholine acetate and naphthalene-2,3-dicarboxaldehyde (Table 1). In addition, the EC treatment led to the presence of 14 oxidative PMs, compared to 7 in the PG/VG group and 8 in the control group. Nearly half of the oxidative PMs in EC have an average saturation above 25%, which is quite a substantial share. These findings indicate a higher burden of both toxic and oxidative protein modifications in the EC-treated cells, suggesting a potentially greater level of cellular stress and damage compared to the other groups. This pattern could plausibly be attributed to the presence of aromatic compounds and nicotine in EC condensates (as both are present). However, based on the chemical modification signatures observed among the toxic/harmful group (Table 1, Figure 4), it is more likely that the modifications stem primarily from aromatic compounds and their combustion products, rather than direct covalent binding by nicotine. In order to definitively disentangle the relative contributions of nicotine versus aromatic compounds in terms of quantitative PM profiling, future experiments should compare e-cigarette liquids containing flavor with and without nicotine.

### 3.4. Determination of Mitochondrial Membrane Potential

**Figure 5:**
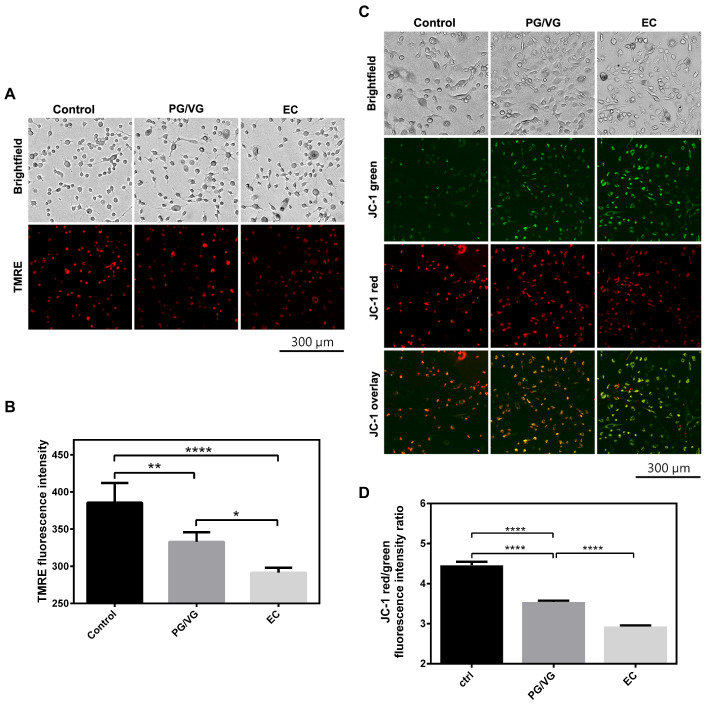
E-cigarette aerosol condensate decreases mitochondrial membrane potential in BEAS-2B cells. (**A**) Potential-dependent staining of mitochondria by TMRE, visualized by ImageXpress^®^ Pico Automated Cell Imaging System. Representative images show an overall reduction in TMRE fluorescence intensity in e-cigarette treatments. (**B**) TMRE fluorescence intensity (a.u.) was quantified using CellReporterXpress software and plotted as mean ± SD. The *p*-values were calculated using one-way ANOVA and Tukey’s post-hoc test. The statistically significant difference between groups is shown as **** (*p* < 0.0001), ** (*p* < 0.01), and * (*p* < 0.05). Results are representative of 3 experiments. Scale bar 300 µm. (**C**) Potential-dependent staining of mitochondria by JC-1 and visualized by ImageXpress^®^ Pico Automated Cell Imaging System. Regions of high mitochondrial polarization are indicated by red fluorescence due to JC-1 aggregate formation, while depolarized regions are indicated by the green fluorescence of JC-1 monomers. (**D**) JC-1 red/green intensity ratio (a.u.) was quantified using Fiji software and plotted as mean ± SEM. The *p*-values were calculated using one-way ANOVA and Tukey’s post-hoc test. The statistically significant difference between groups is shown as **** (*p* < 0.0001). Results are representative of 3 experiments.

Mitochondrial membrane potential was assessed to investigate the effect of EC and PG/VG on mitochondrial function in BEAS-2B cells using the fluorescent cationic dyes TMRE and JC-1. In line with the results of transcriptome and proteome analysis, BEAS-2B cells treated with EC and PG/VG displayed mitochondrial depolarization as evidenced by a significant decrease in TMRE fluorescence intensity (Figure 5A,B). Similarly, JC-1 staining showed a drastic decrease in mitochondrial membrane potential in EC and PG/VG-treated cells, as evidenced by an increase in JC-1 monomers emitting a green fluorescence signal (Figure 5C,D).

### 3.5. E-Cigarette Aerosol Condensate Attenuates Protein Synthesis in BEAS-2B Cells

**Figure 6:**
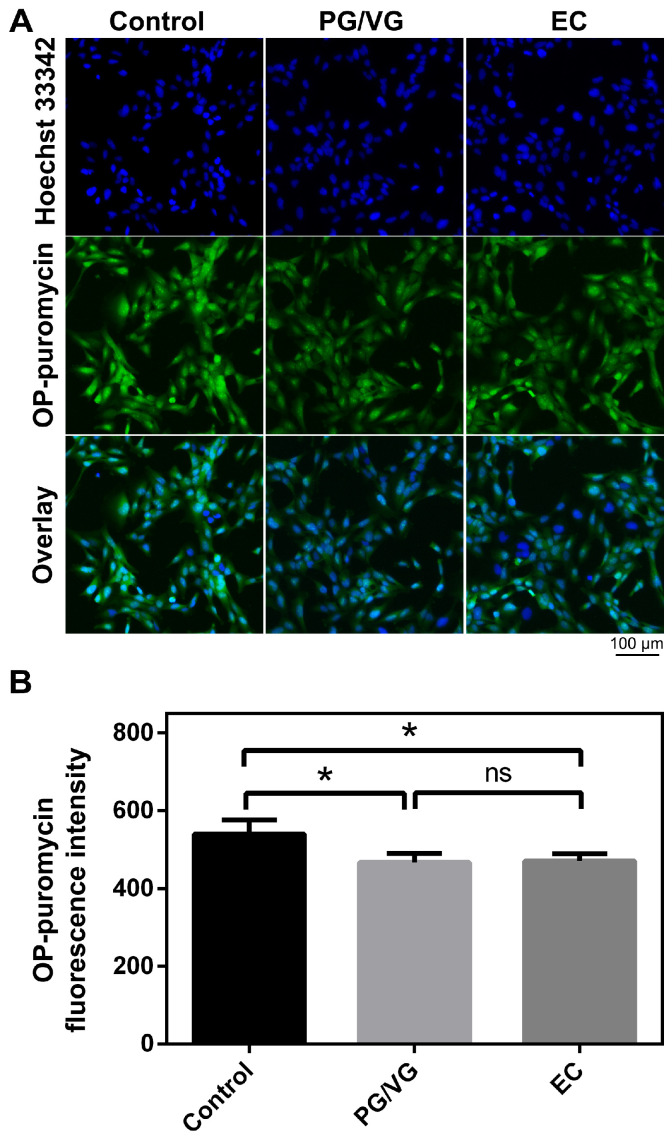
E-cigarette aerosol condensate reduces global protein synthesis in BEAS-2B cells. (**A**) OP-puromycin metabolic labeling of proteins in BEAS-2B cells treated with e-cigarette aerosol condensate visualized by ImageXpress^®^ Pico Automated Cell Imaging System. Nuclei were counterstained with Hoechst 33342. Results are representative of 3 experiments. Scale bar 100 µm. (**B**) OP-puromycin fluorescence intensity (a.u.) was quantified using CellReporterXpress software and plotted as mean ± SD, relative to the untreated control. The *p*-values were calculated using one-way ANOVA and Tukey’s post-hoc test. The statistically significant difference between the groups is shown as * (*p* < 0.05); ns—nonsignificant.

To further evaluate the effect of EC and PG/VG on protein metabolism, cells undergoing translation were labeled using a method based on an alkyne analog of puromycin (OP-puromycin). Analysis of total protein synthesis rate in BEAS-2B cells treated with PG/VG and EC revealed a reduction of protein synthesis compared to the untreated control (Figure 6A,B). The observed difference was statistically significant. Additionally, no difference in total protein synthesis rate between PG/VG and EC treatment groups was observed.

### 3.6. E-Cigarette Aerosol Increases Lysosomal Signal in BEAS-2B Cells

**Figure 7:**
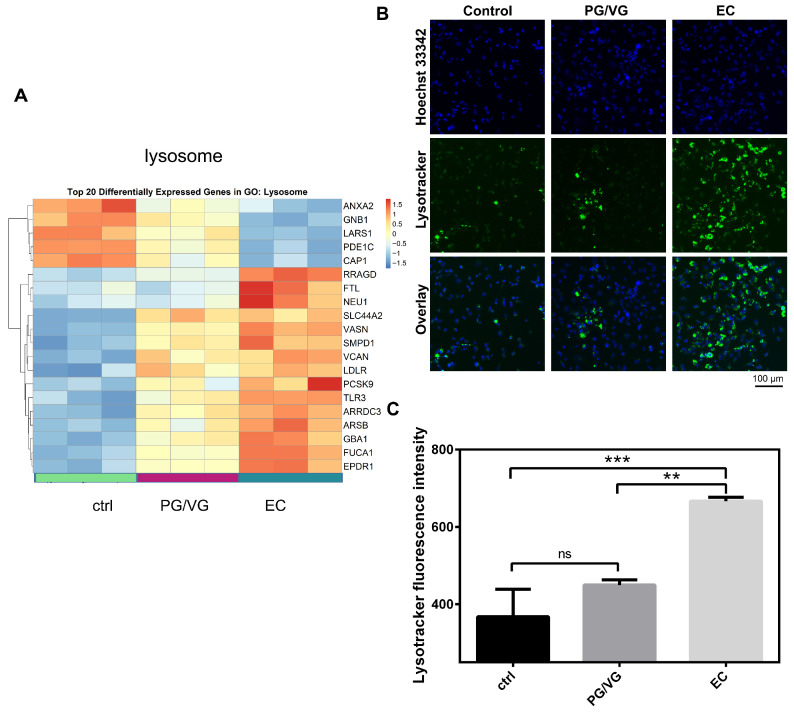
The effects of e-cigarette aerosol condensate on lysosomal signal in BEAS-2B cells. (**A**) Heatmap for the top 20 differentially expressed genes belonging to the Lysosome gene set. (**B**) Lysotracker labeling of BEAS-2B cells treated with e-cigarette aerosol condensate visualized by ImageXpress^®^ Pico Automated Cell Imaging System. Nuclei were counterstained with Hoechst 33342. Scale bar 100 µm. Results are representative of 3 experiments. (**C**) Lysotracker fluorescence intensity (a.u.) was quantified using CellReporterXpress software and plotted as mean ± SD, relative to the untreated control. The *p*-values were calculated using one-way ANOVA and Tukey’s post-hoc test. The statistically significant difference between the groups is shown as ** (*p* < 0.01), and *** (*p* < 0.001); ns—nonsignificant.

To assess lysosomal content in response to EC and PG/VG treatments, we employed Lysotracker, a pH-sensitive dye that preferentially labels acidic compartments within a cell, such as lysosomes. Consistent with the findings of transcriptome analysis (Figure 7A), fluorescence imaging using a lysosome-targeting probe Lysotracker revealed that the lysosomal signal in EC-treated cells was significantly increased compared to the untreated cells, while PG/VG-treated cells showed a slight increase in lysosomal signal (Figure 7A). The fluorescence intensity quantification results are shown in Figure 7B.

### 3.7. E-Cigarette Aerosol Condensate Increases Lipid Droplet Formation in BEAS-2B Cells

**Figure 8:**
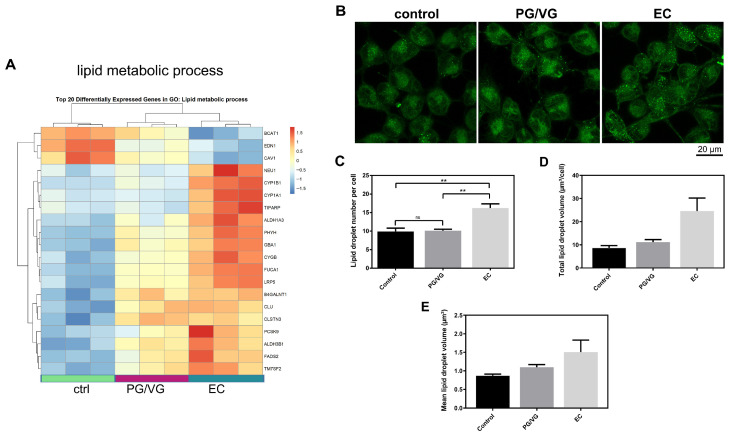
The effects of electronic cigarette aerosol condensate on lipid metabolism in BEAS-2B cells. (**A**) Heatmap for the top 20 differentially expressed genes belonging to the lipid metabolism biological process. (**B**) Representative images of neutral lipid droplets stained with BODIPY™ 493/503. Scale bar 20 µm. Visualized by Leica TCS SP8 confocal microscope. (**C**) Lipid droplet number per cell, (**D**) Total lipid droplet volume per cell, and (**E**) Mean lipid droplet volume were quantified in Fiji software and plotted as mean ± SEM. The *p*-values were calculated using one-way ANOVA and Tukey’s post-hoc test. The statistically significant difference between the groups is shown as ** (*p* < 0.01); ns—nonsignificant. Results are representative of 2 experiments.

BEAS-2B cells were stained with BODIPY™ 493/503 to evaluate the content of neutral lipids following treatment. Consistent with transcriptional changes (Figure 8A), BODIPY™ 493/503 staining of BEAS-2B cells revealed a greater number of lipid droplets in EC-treated cells compared to both the control and PG/VG treatments, indicating enhanced lipid droplet formation (Figure 8B,C). Although total lipid droplet volume per cell and mean lipid droplet volume were elevated in EC-treated cells (Figure 8D,E), these differences did not reach statistical significance, indicating a trend toward increased droplet size and enhanced lipid accumulation.

### 3.8. E-Cigarette Aerosol Condensate Reduces Total Polymerized Actin in BEAS-2B Cells

Phalloidin, a bicyclic peptide that binds with high affinity to F-actin, was used to visualize F-actin and stress fibers in treated cells. Phalloidin staining revealed that EC exposure caused a significant reduction in total polymerized actin per cell compared to control and PG/VG groups (Figure S3A,B). Mean filament intensity per cell showed a non-significant trend toward reduction in EC-treated cells (Figure S3C). F-actin area per cell was unchanged across groups (Figure S3D). In control cells, phalloidin staining showed prominent stress fibers, indicating intact cytoskeletal tension. Importantly, EC exposure significantly reduced total polymerized actin per cell and visibly diminished stress fiber formation, while total F-actin area remained unchanged. PG/VG treatment had no detectable effect. These results indicate that EC reduces filament polymerization without altering the spatial coverage of F-actin within cells, which is in line with the findings of transcriptome analysis of downregulation of actin components.

## 4. Discussion

In the present study, we aimed to evaluate the effect of acute exposure to low doses of e-cigarette aerosol condensate on lung bronchial epithelial cells by comparing the effects of carrier liquid consisting solely of PG and VG with e-cigarettes containing nicotine and flavor. By integrating transcriptomic, proteomic, and cellular assays, this study provides an insight into lung epithelial cell homeostasis after short-term low-level exposure to e-cigarettes, revealing the shared effects of the PG/VG carrier and nicotine/flavored formulations on cellular processes, as well as processes intensified by nicotine/flavored e-cigarettes.

In our transcriptome analysis of e-cigarette effects, omitting an LFC cutoff enabled insight into the full spectrum of subtle gene expression changes induced by e-cigarettes, capturing low-magnitude but biologically relevant alterations, such as the translation process and lipid metabolism, which might be overlooked by higher LFC criteria. We used tobacco-flavored e-cigarettes in this study, reflecting emerging global market trends toward banning non-tobacco and non-mint flavors in e-cigarettes [41,42], and thereby enhancing the translational relevance of our findings. Notably, tobacco-flavored e-cigarettes were also found to exert less pronounced cellular effects on bronchial epithelial cells than flavors such as mint-flavored e-cigarettes [43].

One of the most prominent changes identified in our transcriptome analysis, in both EC and PG/VG-treated cells, was the effect of e-cigarette aerosol condensate on cellular transcription and ribosomal biogenesis. These findings were consistently mirrored at the proteome level, where the ribosome component was found to be decreased in both EC and PG/VG treatments and subsequently confirmed with a cellular assay. Combined with observed chaperone downregulation in our study, these results point to a decline in proteostasis capacity upon e-cigarette exposure. To the best of our knowledge, reports on the effects of e-cigarettes on protein translation remain scarce. It is possible that when applying cutoff criteria in transcriptome analysis, these changes, being that they are subtle, are not being recognized. Our previously published proteome analysis of V79 lung fibroblasts exposed to e-cigarette liquids, the same that were also used here to generate aerosol condensate, identified a reduction in the total number of proteins and downregulation in the translation process, while proteomic analysis of the lung tissue of mice exposed to e-cigarettes found ribosomal proteins to be affected [33,44]. Findings of transcriptomic analysis by Park et al. reported the reduced expression of ribosome genes and a reduction in protein biogenesis in response to vanilla-flavored e-cigarettes [45]. Dysregulation of the translation process in BEAS-2B cells was also observed at the transcriptional level in response to ultrafine particles of diesel and biomass combustion [46]. Therefore, it would be worth examining which component in e-cigarettes is responsible for this effect and comparing the effects of different types and flavors of e-cigarettes.

In addition to cytoplasmic translation downregulation, translational repression was also evident in the downregulation of genes for mitochondrial ribosomes. This downregulation of translation systems within mitochondria was also reflected in the downregulation of cellular components for mitochondria on the proteome level and mitochondrial dysfunction, as evidenced by the decrease in mitochondrial membrane potential after the treatment. Furthermore, observed oxidative protein damage could be both cause and consequence of mitochondrial impairment. In our analysis, mitochondrial function was impaired in response to both EC and PG/VG, suggesting the harmful impact of e-cigarette carriers alone on cellular respiration. Adverse effects of e-cigarettes on mitochondrial function have been previously reported, both in in vitro studies and in e-cigarette users [11,12], and our results point to impaired mitochondrial translation as one of the possible mechanisms by which e-cigarettes induce mitochondrial dysfunction, as well as to this being the shared effect triggered by both PG/VG carriers alone and nicotine and flavor-containing e-cigarettes.

In our transcriptomic analysis, the most significantly upregulated genes, when EC treatment was compared to PG/VG treatment, were cytochrome P450 enzymes *CYP1A1* and *CYP1B1*. In smokers, *CYP1A1* and *CYP1B1* are known to be upregulated by polycyclic aromatic hydrocarbons (PAHs), carcinogens highly abundant in cigarette smoke and also found in e-cigarettes [47,48]. Our results are in line with the recent study presenting the first transcriptome analysis of lung tissue of e-cigarette users that also identified *CYP1A1* as the most upregulated gene compared to non-users [13]. *CYP1A1* and *CYP1B1* were also found to be significantly upregulated in oral keratinocytes exposed to e-cigarette aerosol condensate, and significant upregulation of *CYP1A1* was found in the lungs of rats exposed to e-cigarettes [11,21]. Additionally, the rate of metabolism of a major known tobacco carcinogen, benzo(a)pyrene, to genotoxic products was found to be increased after exposure to e-cigarettes in oral keratinocytes [49]. Enzymes belonging to the cytochrome P450 superfamily also play a major role in lipid metabolism, and *CYP1B1* was found to play a key role in lipid accumulation in alveolar epithelial type II (AT2) cells upon cigarette smoke exposure [36].

Alterations in lipid homeostasis, mainly lipid alterations in AT2 epithelial cells and alveolar macrophages, were also reported after e-cigarette exposure [50,51]. Results of our transcriptome analysis revealed elevated lipid metabolic process, and subsequent imaging revealed increased presence of lipid droplets in EC-treated cells. Presence of macrophages with accumulated excessive lipids, lipid-laden macrophages, was reported in bronchial alveolar fluids (BAL) of e-cigarette users when compared to non-users, as well as in mice chronically exposed to e-cigarettes [50,51]. Chronic e-cigarette vapor exposure was also found to increase intracellular phospholipids in BAL cells, which, combined with the altered macrophages, led to an altered surfactant lipid profile in the lungs, dampened innate immune response and increased susceptibility to inhaled pathogens [51]. In the same study by Madison et al., the authors also reported that alveolar macrophages of mice chronically exposed to e-cigarettes (4-month exposure) presented with an increased number of lysosomes [51]. Our results also showed an elevated lysosomal component in proteomic analysis, which was confirmed by an increase in lysosomal content in BEAS-2B, particularly in EC-treated cells. This lysosomal disruption could reflect changes in cell metabolism and demand for macromolecular or organelle breakdown, which is in line with the observed presence of irreversibly damaged proteins as well as lipid droplets in our study. It is also worth noting that lysosomes are the key accumulation point for fine particulate matter (PM2.5) [52], and e-cigarettes were found to be the source of PM2.5 [53], so their contribution to this effect also needs to be taken into consideration. Further investigation into lysosomal function and autophagy flux, as well as the composition of e-cigarette vapor condensate, is needed to elucidate the mechanism of action on lysosomes. Notably, these perturbations in lipid metabolism and lysosomal abundance observed here after 24 h of e-cigarette exposure mirror those reported in chronic vaping models, highlighting the value of short-term treatments in capturing biologically relevant shifts that align with chronic outcomes.

Protein modification profiles in BEAS-2B cells treated with e-cigarette aerosol condensate revealed an increased number of different chemical derivatives visible as protein modifications in cells treated with EC and PG/VG, suggesting a particularly harmful effect of these treatments, especially EC, as these types of modifications can significantly impact protein folding, functionality, and subcellular localization. Together with the observed translational repression, on both the transcriptomic and proteomic levels, the presence of these protein modifications implies a significant burden on the cellular proteome caused by e-cigarette exposure.

Several chemically induced protein modifications were detected exclusively in EC-treated BEAS-2B cells, revealing the presence of reactive xenobiotic species. The biohazard modification trinitrobenzene, caused by a highly toxic compound [54], was found only in the EC group, consistent with previous observations in V79 lung fibroblasts exposed to e-cigarette liquid [33], and warrants further investigation.

Carbofuran (2,3-dihydro-2,2-dimethyl-7-benzofuranol N-methyl carbamate), a banned insecticide in the US, EU, and Canada due to its high toxicity [55], appeared as a covalent serine modification in EC-treated cells but not in PG/VG or control. This suggests that reactive benzofuran-based electrophiles, likely thermal degradation products of flavoring agents or additives, can acylate hydroxyl groups on serine. This non-enzymatic, xenobiotic-induced PM is distinct from ROS-driven oxidation and indicates that EC aerosols contain reactive aromatic carbamate species capable of targeting critical protein residues. Given the irreversibility of N-methyl carbamate modifications, affected proteins must be degraded, placing additional stress on proteostatic systems [56].

Detection of bis(hydroxyphenylglyoxal) arginine in EC-treated BEAS-2B cells indicates the formation of reactive α-dicarbonyl species, particularly glyoxal and its aromatic derivatives. Glyoxal, a highly reactive 1,2-dialdehyde, promotes arginine-selective glycation and the formation of advanced glycation end products (AGEs) [57].

These PMs arise from covalent reactions between glyoxal derivatives and the guanidino group of arginine, forming stable adducts that disrupt protein structure and function. Such modifications may compete with physiological arginine PMs and interfere with signaling pathways linked to inflammation and cellular stress. AGEs formed by glyoxal and related species are implicated in oxidative stress-related diseases, including diabetes [58], atherosclerosis [59,60], and neurodegenerative disorders [61].

A PM corresponding to 5-dimethylaminonaphthalene-1-sulfonyl (dansyl) was identified on valine residues. Structurally analogous to dansyl chloride, this modification forms stable sulfonamide adducts with primary and secondary amines under physiological conditions [62]. Its presence suggests that electrophilic aromatic sulfonyl compounds generated by EC aerosols can irreversibly modify lung epithelial proteins through aromatic sulfonylation [62].

O-diisopropylphosphorylation, a chemically induced phosphorylation-like PM, was also observed following EC treatment. This covalent adduct, associated with toxic organophosphorus exposure, can promote protein crosslinking and has been linked to neurotoxicity [63].

N-succinimidyl-2-morpholine acetate (SMA), typically a synthetic crosslinker used in vitro, targets primary amines on lysine side chains and N-termini. Its detection in EC-treated BEAS-2B cells suggests that aerosol-derived succinimidyl-like compounds can induce non-enzymatic covalent modifications resembling NHS ester-mediated lysine acetylation.

Finally, naphthalene-2,3-dicarboxaldehyde, a reactive aromatic dialdehyde, was identified. Known to form fluorescent isoindole derivatives with lysine and N-termini, its presence supports the idea that EC aerosols contain volatile aromatic aldehydes capable of covalent protein labelling [64].

The appearance of 2,3-dihydro-2,2-dimethyl-7-benzofuranol N-methyl carbamate as a covalent modification on serine residues in BEAS-2B cells treated with EC, but not in PG/VG-treated cells and control, strongly suggests that reactive benzofuran-based electrophiles, possibly thermal degradation products of flavoring agents or additives, are capable of acylating hydroxyl groups on serine. This PM reflects a non-enzymatic, xenobiotic-induced modification distinct from ROS-driven oxidation. In the context of exposure to e-cigarette aerosols, this PM indicates that the aerosol contains reactive aromatic carbamate species capable of targeting critical protein residues. Because covalent N-methyl carbamate modifications are typically irreversible, modified proteins must be turned over, placing additional burden on proteostatic systems. For example, increased and dysregulated proline oxidation to pyrrolidinone is associated with impaired protein function and linked to diseases such as cancer and other stress-related conditions [65].

It needs to be noted that chemical modifications commonly detected by mass spectrometry and labelled in the Unimod database as CD are typically associated with intentional derivatization for analytical purposes. However, in the context of protein modification analysis following exposure to toxic treatments, similar or even identical modifications may arise unintentionally due to environmental chemical reactions. This overlap can lead to misidentification or misclassification of certain protein modifications, where treatment-induced changes are mistakenly attributed to known reagents or deliberate processes. Beyond database overlap, there is also the possibility of mass shift isomers, where distinct chemical modifications can produce similar mass changes, and we acknowledge this as a limitation of current PM analysis despite applying stringent scoring and manual validation.

To mitigate this ambiguity, it would be most optimal to perform de novo PMs analysis alongside open, unspecified database-driven matching, which is preferable to a specified search, as the latter may overlook context-specific modifications [33,66]. In summary, while protein identification has been mastered long ago by bottom-up mass-spectrometry-based proteomics, global quantitative profiling of protein modifications is still under improvement due to challenges associated with their reliable and confident identification and validation [67].

The disruption of proteostasis, mitochondrial function, and lipid metabolism are processes known to contribute to chronic lung diseases, including chronic obstructive pulmonary disease (COPD) and lung cancer. This, together with observed upregulation of xenobiotic-metabolizing enzymes and accumulation of toxic protein modifications, supports concerns that e-cigarette exposure may initiate pathogenic pathways similar to those induced by conventional cigarette smoke. The early molecular changes detected in our study, particularly irreversible protein modifications, could serve as potential clinical biomarkers of subclinical injury caused by e-cigarette use. One of the observed protein modifications is of particular potential interest. 3-nitrotyrosine (3-NT), a biomarker of nitroxdative stress, was found to be elevated in airways of patients with chronic lung diseases (such as COPD and asthma) and also to be induced by smoking [68,69]. Although we did not detect 3-NT in our samples, we have observed the presence of 2-aminotyrosine (2-AT) after exposure to EC. The presence of 2-AT can be a consequence of the reduction of the nitro group on 3-NT, and the detection of 2-AT can indicate nitroxidative stress even in the absence of detectable 3-nitrotyrosine. Given its greater chemical stability, 2-AT may represent a persistent footprint of aerosol-induced protein damage and warrants further evaluation as a potential biomarker of e-cigarette exposure.

There are several limitations of this study that should be mentioned. First, the experiments were conducted using a single immortalized bronchial epithelial cell line, which, although widely used, does not fully capture the cellular diversity and complexity of the human airway epithelium. Future studies incorporating primary human bronchial epithelial cells, air–liquid interface cultures, or co-culture systems would improve physiological relevance. Second, we used acute, short-term treatment with e-cigarette aerosol condensates, which has allowed us to capture early molecular events. Long-term exposure studies are needed to determine whether the observed transcriptomic and proteomic alterations persist, adapt, or worsen over time. Finally, although we identified numerous protein modifications associated with e-cigarette exposure, functional validation of their direct biological consequences was beyond the scope of this study. Future work should aim to link specific environmentally induced protein modifications to altered cellular function and disease-relevant outcomes.

In conclusion, our findings revealed that non-cytotoxic exposure to e-cigarette aerosol disrupts proteostasis and cellular fitness in BEAS-2B cells, as evidenced by changes in cellular translation, lipid metabolism, mitochondrial function, and protein modifications. The presence of most of these changes corroborates findings from chronic exposure models and from e-cigarette users, underscoring their early onset and potential persistence in e-cigarette-induced cellular stress. Our results show that key cellular processes such as protein translation and mitochondrial function are affected by both carrier liquids alone as well as by e-cigarettes containing nicotine and flavor, pointing to the harmful effect of base components beyond nicotine or flavor. Ultimately, these results raise concerns about the potential of e-cigarettes to prime lung epithelial cells for long-term dysfunction and disease susceptibility.

## Figures and Tables

**Table 1:** Relative protein modification profiling control and treated BEAS-2B cell proteomes.

Modification Type	Unimod Label	Average Δm Shift (a.u.)	Control (CTRL)		PG/VG		EC	
			Ratio	%	Ratio	%	Ratio	%
**OXIDATIVE MODIFICATIONS**								
**I Carbonylation due to direct oxidation of Lys, Thr and Pro amino acid side chains**								
Proline oxidation to pyrrolidinone	CD	−30.0260	N/A	0	N/A	0	∞	100
Pyrrolidone from Pro	CD	−28.0101	N/A	0	N/A	0	∞	14
2-amino-3-oxo-butanoic_acid from Thr	P/CD	−2.0159	1.0 ± 1.2	6 ± 7	0.5 ± 0.5	4 ± 3	0.03 ± 0.01	0.5 ± 0.2
Lysine oxidation to aminoadipic semialdehyde	P/CD	−1.0311	N/A	0	1.0 ± 0.1	43 ± 37	2.2	62
**II Carbonylation due to Michael addition reaction of α,β-unsaturated aldehydes derived from lipid peroxidation**								
Levuglandinyl-lysine anhyropyrrole adduct	P/CD	298.4192	1.0 ± 0.1	39 ± 4	1.1 ± 0.5	34 ± 12	1.7 ± 1.4	24 ± 10
Levuglandinyl-arginine lactam adduct	P/CD	290.3939	N/A	0	∞	100	N/A	0
**III Carbonylation events due to AGEs formation**								
Condensation product of glucosone on Arg	CD	160.12470	N/A	0	1.0	3	1.6	56
Carboxyethyl (H)	CD	72.06270	N/A	0	N/A	0	∞	0.4
**Other direct oxidative modifications**								
Ethanolation (K and R)	CD	44.0526	N/A	0	N/A	0	∞	12 ± 6
Dihydroxy (K)	P	31.9988	1	21	0	0	0	0
Dihydroxy (R and/or P)	P	31.9988	N/A	0	N/A	0	∞	97 ± 3
Met double oxidation (sulphone)	A	31.9988	1.0 ± 0.7	4	0	0	0.2	2
Met single oxidation (sulfoxide)	P/CD	15.9994	1.0 ± 0.2	28 ± 7	1.0 ± 0.5	23 ± 6	0.7 ± 0.1 *	23 ± 6
His and Trp oxidation	A	15.9994	1.0 ± 0.5	8 ± 2	1.3 ± 0.4	4 ± 2	0.7 ± 0.1	8 ± 1
Oxidation or Hydroxylation of Asn	P	15.9994	1	38	0	0	0	0
Tyrosine oxidation to 2-aminotyrosine	CD	15.0146	N/A	0	N/A	0	∞	5
Proline oxidation to pyroglutamic acid	A	13.9835	1	3	0	0	0	0
Tryptophan oxidation to oxolactone	CD	13.9835	N/A	0	N/A	0	∞	25
**PHYSIOLOGICAL MODIFICATIONS (PTMs)**								
Acetylation (K)	P/CD	42.0367	1.0 ± 1.0	93 ± 12	1.4 ± 0.4	73 ± 15	0.7 ± 0.5	67 ± 2
Acetylation (Protein N-term)	P/CD	42.0367	1.0 ± 0.1	94 ± 2	1.0 ± 0.1	91 ± 3	1.2 ± 0.3	93 ± 2
Methylation(KR)	P	14.0266	1.0 ± 0.2	86 ± 13	3.4 ± 1.5	13 ± 10	1.3 ± 1.6	60 ± 49
Biotinylation (GNLK)	P	226.2954	1.0 ± 0.8	22 ± 16	1.5 ± 0.2	22 ± 8	1.0 ± 1.2	49 ± 15
Dimethylation (KR)	P	28.0532	1.0 ± 0.2	86 ± 13	2.1 ± 0.2	100 ± 0	1.5 ± 1.4	92 ± 5
Diethylation	P	56.1063	1.0 ± 0.3	41 ± 29	2.2	51	1.6 ± 0.7	42 ± 8
Heme (H)	P	616.4873	1.0 ± 0.6	63 ± 19	1.4 ± 0.6	70 ± 1	1.6 ± 0.6	62 ± 1
Dehydration (S,T,N,Q,Y)	P	−18.0153	1.0	30	2.2 ± 0.4	37 ± 11	0.7	18
Myristoylation (GCK)	P	210.3556	1.0 ± 0.1	52 ± 68	4.3 ± 4.3	100 ± 0	4.6	100
Dehydration (D)	P	−18.0153	1.0 ± 0.3	3 ± 1	0	0	0	0
Hydroxyheme E	P	614.4714	N/A	0	∞	0.4	N/A	0
Phosphorylation (STY)	P	79.9799	1.0	9	5.1	43	4.3 ± 1.0	44 ± 2
Palmitoylation (KT)	P	238.4088	1.0 ± 0.8	52 ± 67	0	0	0	0
Decanoyl (S)	P	154.2493	N/A	0	N/A	0	∞	5
Octanoyl (T)	P	126.1962	1.0 ± 0.5	5 ± 2	1.1 ± 0.3	4 ± 1	0.9 ± 0.0	5 ± 1
Lipoyl (K)	P	188.3103	N/A	0	∞	100	∞	100
O3-(riboflavin) phosphoryl (T)	P	438.3285	1.0	1	0	0	0	0
Triglutamyl on Glu	P	387.3419	1.0	5	0	0	0	0
Phosphopantetheine (S)	P	340.333	1.0	98	0	0	0	0
Deamidation (R)	P	−0.9848	1.0 ± 1.2	43	0	0	0	0
N-acetyl neuraminic acid (T)	P	291.2546	1.0	19	0	0	0.6	17
Sulfation (S)	P	80.0632	N/A	0	N/A	0	∞	1
Pyridoxal phosphate (K)	P	229.1266	N/A	0	∞	100	N/A	0
Hypusine (K)	P	87.1204	1.0	31	0	0	0	0
**TOXIC/BIOHAZARD MODIFICATIONS (Chemical derivatives-CD)**								
Tri nitro benzene (R)	CD	211.0886	N/A	0	N/A	0	∞	5
2 3-dihydro-2 2-dimethyl-7-benzofuranol N-methyl carbamate (S)	CD	57.0153	N/A	0	N/A	0	∞	48
Bis(hydroxphenylglyoxal) arginine	CD	282.2476	N/A	0	N/A	0	∞	6
5-dimethylaminonaphthalene-1-sulfonyl (V)	CD	233.2862	N/A	0	N/A	0	∞	0.3
O-Diisopropylphosphorylation (AV)	CD	164.1394	N/A	0	N/A	0	∞	100
N-Succinimidyl-2-morpholine acetate (LIP)	CD	127.1412	N/A	0	∞	48 ± 23	∞	70
Naphthalene-2 3-dicarboxaldehyde (LNW)	CD	175.1855	N/A	0	N/A	0	∞	50 ± 70
Acetaldehyde +26 (H)	CD	26.0373	N/A	0	∞	100	N/A	0
Chlorination of tyrosine residues	CD	34.4451	N/A	0	∞	100	N/A	0
Fluorination (FWYA)	CD	17.9905	N/A	0	∞	1	N/A	0
**ENVIRONMENTAL MODIFICATIONS (Chemical derivatives-CD)**								
Ubiquitination (K)	CD	383.446	1.0 ± 0.9	59 ± 36	1.2 ± 0.2	52 ± 50	1.3 ± 0.8	41 ± 13
Phenethyl isothiocyanate (ES)	CD	163.2395	N/A	0	∞	100	N/A	0
Nucleophilic addition to cytopiloyne (KT)	CD	362.3738	N/A	0	∞	57 ± 61	N/A	0
Methylmalonylation (S)	CD	100.0728	N/A	0	∞	34	N/A	0
Arginine replacement by Nitropyrimidyl ornithine	CD	81.0297	N/A	0	∞	13	N/A	0
Addition of DFDNB crosslinker (K)	CD	164.0752	N/A	0	∞	67 ± 11	N/A	0
Beta-methylthiolation (C)	CD	46.0196	N/A	0	∞	100	N/A	0
Aminoethylcysteine (T)	CD	59.1334	N/A	0	∞	5.0 ± 0.4	N/A	0
Iminobiotinylation (T)	CD	225.3106	N/A	0	∞	8 ± 3	N/A	0
Piperidination (EY)	CD	68.1170	N/A	0	∞	3	∞	47 ± 5
EDT-maleimide-PEO-biotin (T)	CD	601.8201	N/A	0	∞	48	N/A	0
Maleimide (K)	CD	97.0721	N/A	0	N/A	0	∞	5
**ARTEFACT MODIFICATIONS**								
Amidation (any C term)	A	−0.9848	1	86	1.1 ± 0.6	71 ± 41	0	0
Carbamoylation (KR)	A	43.0247	1	1	2.5	1	1.2 ± 1.0	1 ± 1
Deamidation (NQ)	A	0.9848	1.0 ± 0.1	10 ± 3	3.8 ± 4.5	40 ± 18	1.1 ± 0.2	14 ± 3
Formylation (KR)	A	28.0101	1.0 ± 1.3	28 ± 35	1.1 ± 0.9	66 ± 19	2.0 ± 0.3	55 ± 15
Pyro-glu from Q	A	−17.0305	1.0 ± 0.5	14 ± 5	1.1 ± 0.3	25 ± 14	1.0 ± 0.4	26 ± 2
Pyro-glu from E	A	−18.0153	1.0 ± 0.4	4 ± 5	0.2 ± 0.1	2.0 ± 0.4	0.3 ± 0.3	23 ± 16

* Ratio: sum of areas under extracted ion chromatography (XIC) curves of all modified peptides bearing the same modification type divided by the sum of XIC curve areas of modified peptides from the control group. CD—chemical derivative modification, P—physiological modification (posttranslational modification (PTM) or PTM-like protein modification), A—artefact protein modification, Δm—delta mass (e.g., change in the initial and resulting mass after the modification introduction); N/A: not applicable since modified peptide form does not exist (e.g., only unmodified form of peptide is present). ∞: when dividing an integer by 0, as in the case when expressing a ratio where the XIC value of the control modified peptide is 0.

## Data Availability

RNA-seq data have been deposited at NCBI’s Sequence Read Archive (SRA) with SRA BioProject accession number PRJNA1198719 and Gene Expression Omnibus (GEO) with GEO series accession number GSE315683. The mass spectrometry proteomics data have been deposited to the ProteomeXchange Consortium via the PRIDE partner repository with the dataset identifier PXD072831 and 10.6019/PXD072831.

## Supplementary Materials

The following supporting information can be downloaded at: https://www.mdpi.com/article/10.3390/cells15060525/s1, Figure S1: Effects of e-cigarette aerosol condensate on cell viability in BEAS-2B cells, as determined by MTT assay (24 h treatment). Data shown as % of control and are the mean ± SEM of 3 independent replicates. *p*-values were calculated using an unpaired *t*-test with Welch’s correction; Figure S2: Gene Ontology enrichment analysis of differentially expressed genes (DEGs) unique to 3 different comparisons (ctrl vs. PG/VG, ctrl vs. EC, PG/VG vs. EC); Figure S3: Electronic cigarette aerosol condensate reduces total F-actin in BEAS-2B cells. (A) F-actin was stained with Alexa Fluor™ 555 phalloidin and visualized by Leica DMRB microscope. Representative images show diminished F-actin in EC-treated cells compared to control and PG/VG. Scale bar = 100 µm. (B) Total F-actin per cell (integrated density, a.u.) was quantified using Fiji software and plotted as mean ± SEM. The *p*-values were calculated using one-way ANOVA and Tukey’s post-hoc test. The statistically significant difference between the groups is shown as *** (*p* < 0.001). ns—nonsignificant. (C) Mean F-actin intensity per cell (a.u.) and (D) Mean F-actin area per cell (a.u.) were quantified using Fiji software and plotted as mean ± SEM. Results are representative of 2 experiments.; Table S1: List of differentially expressed genes (DEGs) for all 3 comparisons (ctrl vs. PG/VG, ctrl vs. EC, PG/VG vs. EC); Table S2: The proteomic, label-free quantitative findings of differentially expressed proteins (DEPs).

## Abbreviations

The following abbreviations are used in this manuscript:

A	Artefact
AT2	Alveolar epithelial type II cells
a.u.	Arbitrary units
BAL	Bronchial alveolar fluid
BP	Biological process
CD	Chemical derivative
CC	Cellular compartment
CTRL	Control
DEGs	Differentially expressed genes
DEPs	Differentially expressed proteins
DMSO	Dimethyl sulfoxide
EC	Electronic cigarette containing propylene glycol, glycerol, nicotine and flavor
FDR	False discovery rate
GO	Gene ontology
LFC	Log fold change
LFQ	Label free quantification
MTT	Thiazolyl Blue Tetrazolium Blue
MF	Molecular function
nLC-MS/MS	Nano liquid chromatography coupled to high resolution tandem mass spectrometry
P	Physiological modification
PBS	Phosphate-buffered saline
PG	Propylene glycol
PM	Protein modification
PM2.5	Fine particulate matter
PTM	Post translational modification
SDS PAGE	Sodium dodecyl sulphate-polyacrylamide gel electrophoresis
TMRE	Tetramethyl rhodamine ethyl ester
VG	Vegetable glycerin
