# Supplementary figures and images for: Acute E-Cigarette Aerosol Condensate Exposure Disrupts the Transcriptome and Proteome Profiles of Human Bronchial Epithelial BEAS-2B Cells
Source: Cells. 2026 Mar 16;15(6):525. doi: 10.3390/cells15060525 (PMC13025378; doi:10.3390/cells15060525)

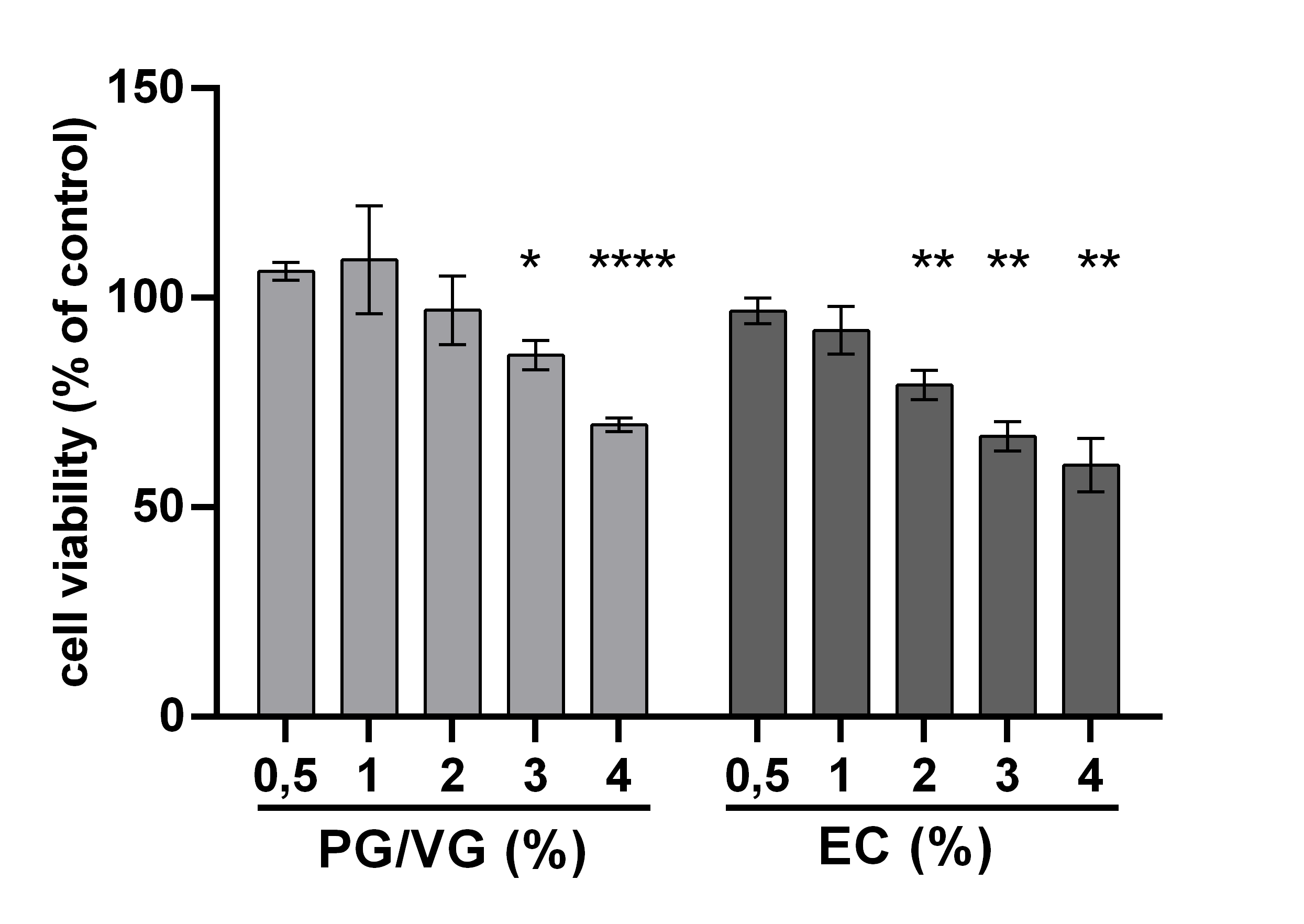

Supplement: Supplementary file 1 [file cells-15-00525-s001.zip › Figure S1 Trifunovic et al.tif]

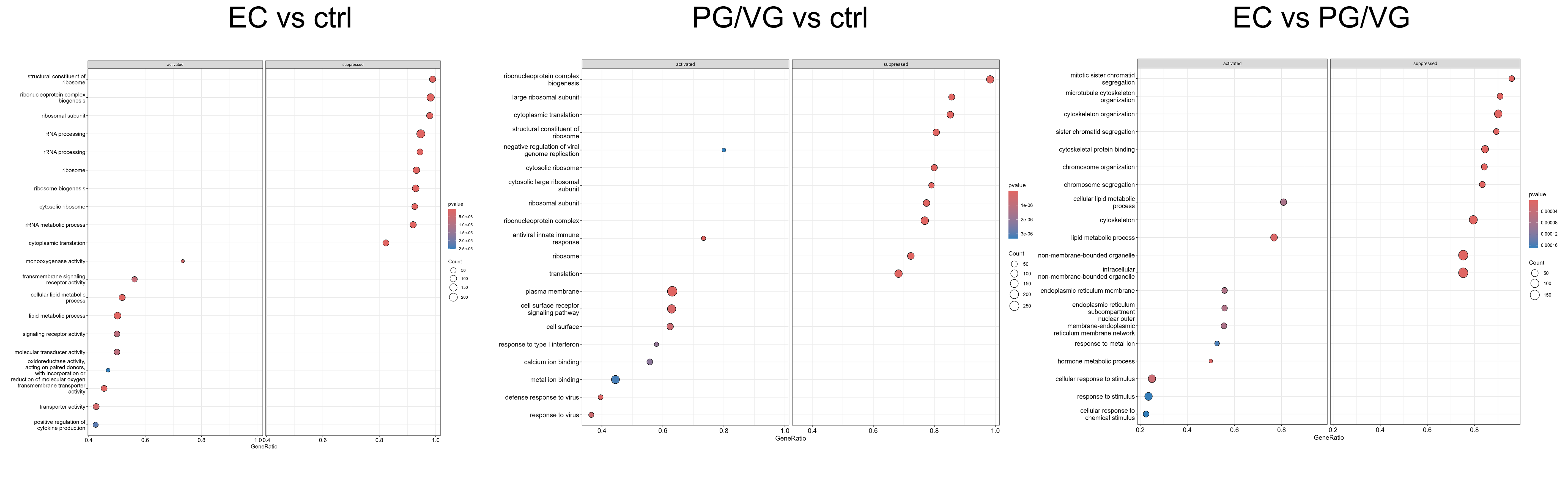

Supplement: Supplementary file 1 [file cells-15-00525-s001.zip › Figure S2 Trifunovic et al.tif]

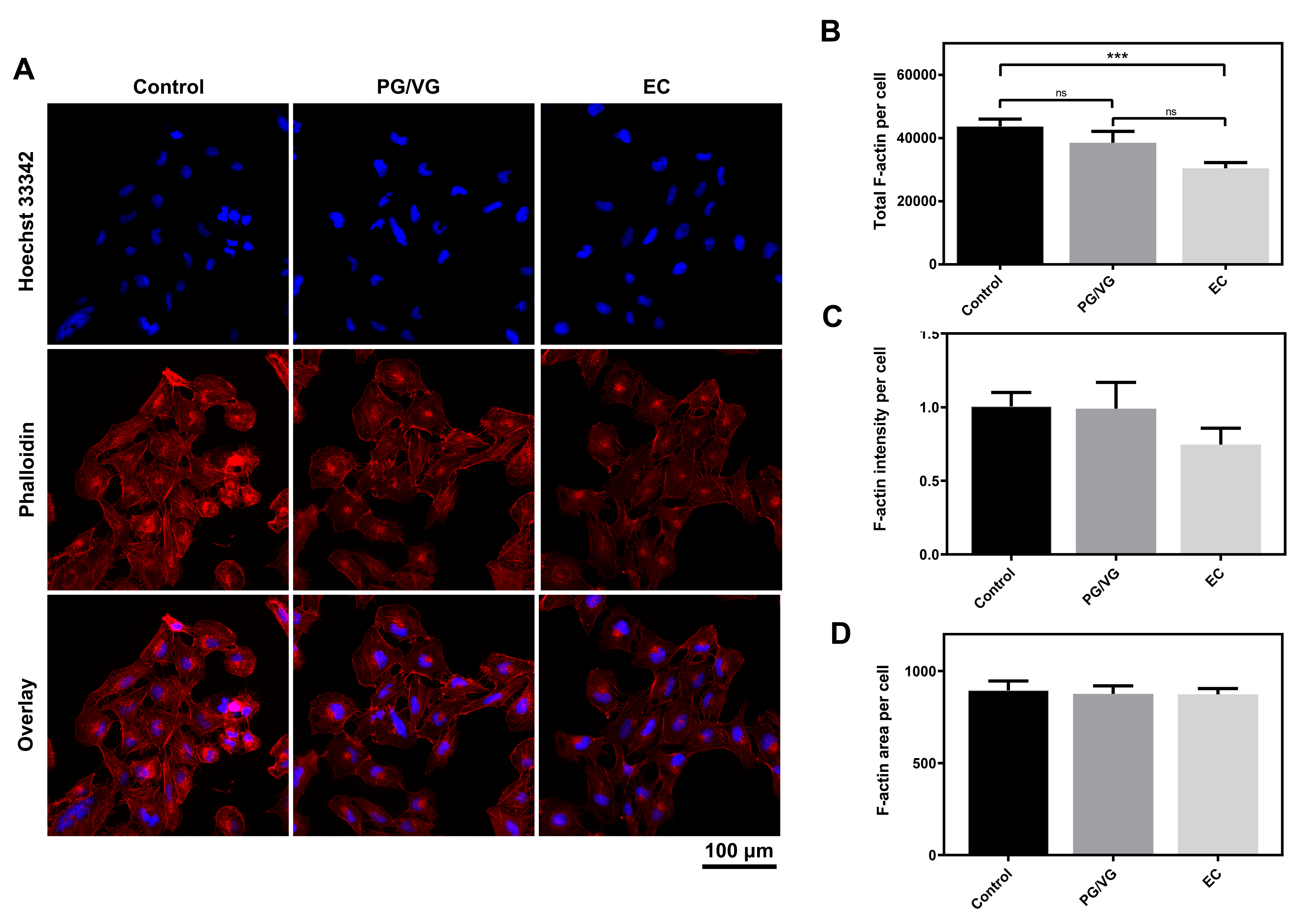

Supplement: Supplementary file 1 [file cells-15-00525-s001.zip › Figure S3 Trifunovic at al revised.tif]
